# Improving quality of intrapartum and immediate postpartum care in public facilities: experiences and lessons learned from Rajasthan state, India

**DOI:** 10.1186/s12884-022-04888-5

**Published:** 2022-07-23

**Authors:** Yashpal Jain, Tarun Chaudhary, Chandra Shekhar Joshi, Manish Chotiya, Bijali Sinha, Tapas Sadasivan Nair, Ashish Srivastava, Vinod Kumar SV, Abhinav Agrawal, Vineet Srivastava, Dinesh Baswal, Kamlesh Lalchandani, Hemang Shah, Gulnoza Usmanova, Bulbul Sood, Vikas Yadav, Somesh Kumar

**Affiliations:** 1 Jhpiego, Jaipur, Rajasthan India; 2 Department of Medical, Health and Family Welfare, Maternal Health, Rajasthan, India; 3 Jhpiego, New Delhi, India; 4 Ministry of Health and Family Welfare, Maternal Health Division, New Delhi, India; 5 The Children’s Investment Fund Foundation, New Delhi, India

**Keywords:** Intrapartum care, Postpartum care, Maternal health, Newborn health, Quality improvement

## Abstract

**Background:**

In spite of considerable improvement in maternal and neonatal outcomes over the past decade in India, the current maternal mortality ratio and neonatal mortality rate are far from the Sustainable Development Goal targets due to suboptimal quality of maternity care. A package of interventions for improving quality of intrapartum and immediate postpartum care was co-designed with the Ministry of Health as the Dakshata program and implemented in public sector health facilities in selected districts in the state of Rajasthan of India since June 2015. This article describes the key strategies, interventions, results and challenges from four years of Dakshata program implementation.

**Methods:**

We have conducted secondary analysis of program data (government data) collected from 202 public facilities across 20 districts of Rajasthan state. The data collected between June–August 2015 (baseline) and the data collected between May-August 2019 (latest) were analyzed. The data sources included: facility assessments, service statistics, monthly progress reports.

**Results:**

During the period of program implementation, there were 17,94,249 deliveries accounting for 70% of institutional deliveries in intervention districts. As a result of the intervention, there was a notable increase in competency of health care providers, availability of essential resources, achievement of labour room standards and adherence to evidence-based clinical standards. We also observed reductions in the proportion of referrals for pre-eclampsia/eclampsia, postpartum hemorrhage and neonatal asphyxia by 11, 8 and 3 percentage points respectively. Similarly, data revealed a reduction in stillbirth rates in Dakshata intervention facilities (19.3 vs 15.3) compared to non-Dakshata facilities (21.8 vs 18).

**Conclusions:**

Our experience and findings indicate that the quality of intrapartum and immediate postpartum care can be improved in low- and middle-income countries with the approach presented in this paper.

**Supplementary Information:**

The online version contains supplementary material available at 10.1186/s12884-022-04888-5.

## Introduction

One of the Sustainable Development Goals (SDG) is to reach fewer than 70 maternal deaths per 100,000 live births and neonatal deaths fewer than 12 per 1,000 live births globally by 2030 [1]. In spite of considerable improvement in maternal and neonatal outcomes over the past decade in India [2], the current maternal mortality ratio (122 deaths per 100,000 live births) [3] and neonatal mortality rate (33 deaths per 1,000 live births) [4] are far from the SDG’s targets.

As a result of various national schemes [5–7], rates of institutional delivery increased significantly (88.6 vs. 78.9%) in the last few years in India [8]. However, maternal and neonatal outcomes did not show commensurate improvement for the same time period [9], indicating issues with quality of maternity care. Many factors influence the quality of intrapartum and immediate postpartum care: availability of resources (both human and material), skills of health care workers, compliance to evidence-based guidelines and an enabling environment [10–12].

To address these issues, Jhpiego, in collaboration with the Government of Rajasthan and with funding support from Children’s Investment Fund Foundation (CIFF), implemented an intrapartum and immediate postpartum quality improvement initiative [13]. The World Health Organization Safe Childbirth Checklist (SCC) was part of this quality improvement initiative which aimed at improving health providers’ adherence to 28 key clinical practices during labour and immediate postpartum period [13]. Furthermore, an independent evaluation of this initiative showed 11% reduction in facility-based mortality (stillbirths and very early neonatal deaths) [14]. As a result, the Ministry of Health and Family Welfare (MoHFW), Government of India, scaled up this initiative throughout the country under the name “Dakshata” (adroitness) [15].

In this article, we describe the process of implementing Dakshata initiative in selected public hospitals in Rajasthan state of India. We report the key strategies, interventions, results and challenges from four years of Dakshata program implementation. To our knowledge, this is one of the largest efforts to implement and evaluate a quality improvement initiative in a real-world setting in low- to middle-income countries.

### Description of the Intervention

#### Program Area

Rajasthan is the largest state in western India, with 84% institutional deliveries, out of which 63% occur in public facilities; the maternal mortality ratio is 164 per 100,000 live births [16] and infant mortality rate is 37 per thousand live births [17].

As a strategic decision (Table 1), the Dakshata program targeted facilities with more than 50 deliveries per month, mainly district hospital, sub-district hospitals, and community health centers. It enabled us to cover ~ 80% of deliveries happening in the districts and efficiently utilize resources.

**Table 1:** Dakshata strategy

The overall goal of Dakshata program is to improve the quality of maternal and newborn care during the intrapartum and immediate postpartum period, through providers who are competent and confident^1^ The major objectives of the initiative are:	
1. To strengthen the competency of providers of the labour room, including medical officers, staff nurses, and auxiliary nurse midwives to perform evidence-based practices as per the established labour room protocols and standards 2. To implement enabling strategies to ensure transfer of learning towards improved adherence to evidence-based clinical practices 3. To improve the availability of essential supplies and commodities in the labour room and the postpartum wards 4. To improve accountability of service providers through improved recording, reporting and	

**Fig. 1:**
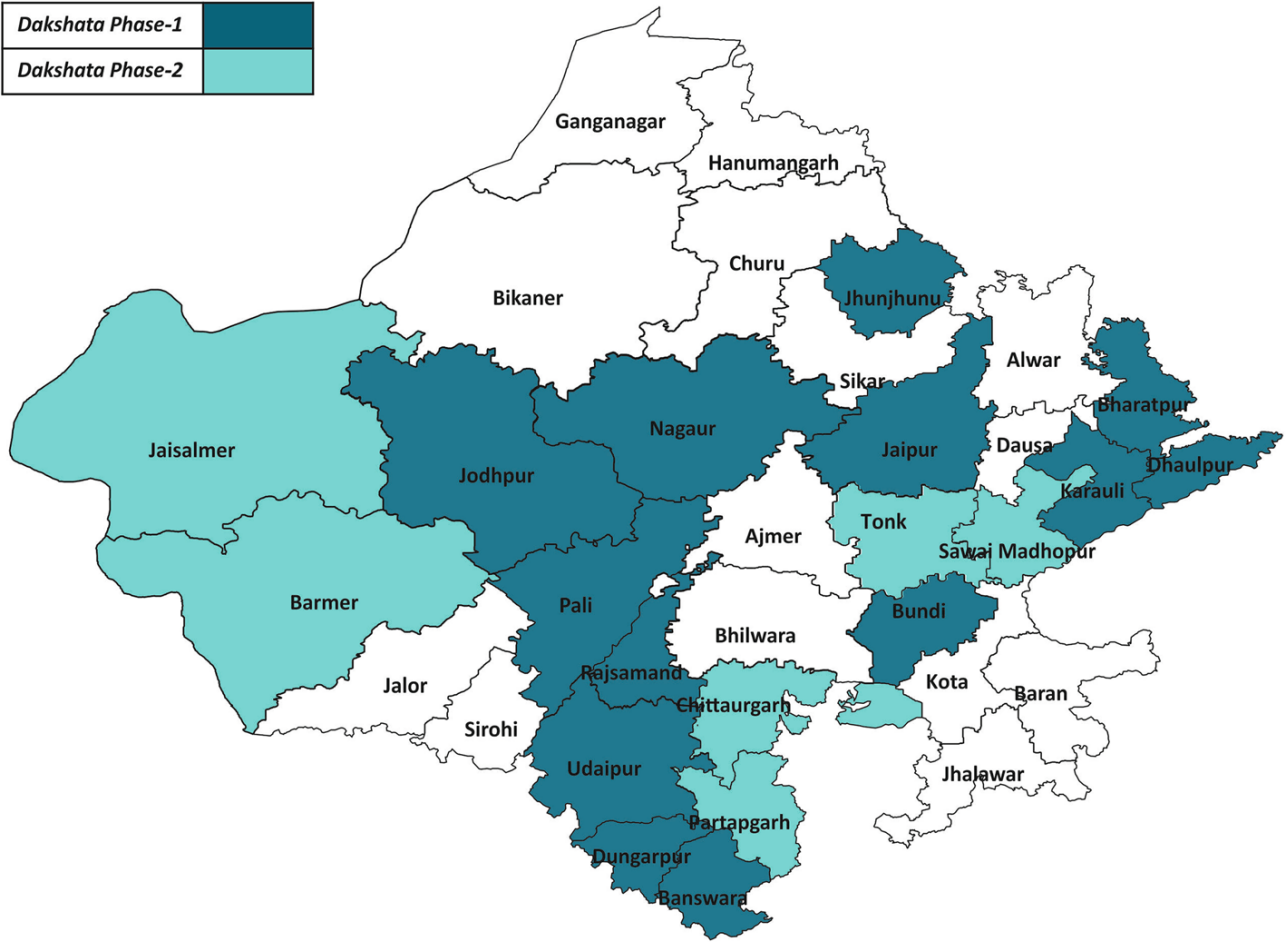
Intervention districts in the state of Rajasthan

The first phase of the program was implemented in 147 facilities in 13 districts, beginning in 2015. In a second phase in 2017, 55 facilities in seven more districts were targeted. In October 2018, the Government of Rajasthan scaled up the program across all 34 districts in the state (Fig. 1 ), that includes 525 facilities.

#### Program activities

**Fig. 2:**
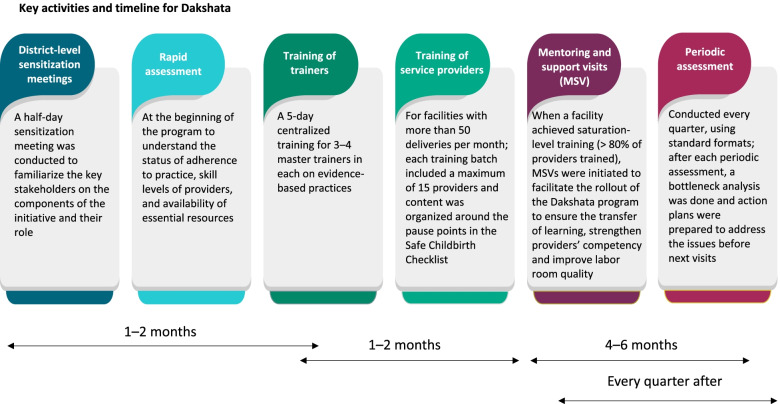
Key activities and timeline for Dakshata

Figure 2 summarizes the key activities and timelines of Dakshata program aligned with the objectives of the program and existing Government of India guidelines [15, 18–20].

The health care providers were trained on clinical skills such as management of normal labour and initial management of maternal and neonatal complications. This training also included training staff to differentiate fresh stillbirth and immediate neonatal death. Additionally, the providers were again oriented on clinical skills, differentiation of fresh stillbirth during mentoring and supporte visits (MSVs). Periodic assessments and refreshment trainings were provided as needed.

Overall, nine training of trainers for the duration of 5 days and 279 trainings for health care providers for the duration of 3 days with 10–15 people per group were conducted. The tools used for knowledge, skills and competency were as per Dakshata package [21]. The skill assessment took on average 2 h. Skills were also subsequently assessed during periodic assessments and MSVs.

## Methods

### Study design and study setting

We conducted secondary analysis of program data (government data) collected from 202 public facilities across 20 districts of Rajasthan state (Table 2) covering 80% of deliveries in intervention districts.

**Table 2:** Type and number of intervention facilities

	Type of the facility				Total
Number	District hospital	Sub divisional hospital	Community health center	Primary health center	
	19	13	153	17	202

This de-identified dataset was provided by the Government of Rajasthan. The data collected between June–﻿August 2015 was considered as a baseline and the data collected between May–August 2019 was considered as the latest assessment. The study was performed in accordance with ethical principles outlined in the World Medical Association Declaration of Helsinki where privacy and confidentiality of personal information was assured by getting access to de-identified data. The study was reviewed and approved by the Government of Rajasthan. The Johns Hopkins Bloomberg School of Public Health Institutional Review Board (IRB) reviewed the activities and determined them to be not human subjects research and thus not requiring IRB oversight (IRB No: 00005529).

### Study tools and data collection

The following tools used for data collection were approved by Government of India as part of monitoring activities under Dakshata program [21].

### Primary data

#### Facility assessments

A rapid assessment was conducted by program officers with medical backgrounds in all program sites before roll out of Dakshata program. The assessment took 4–6 h to complete. The same tool was used for quarterly periodic assessments (Table 3). All the tools contained verification criteria to avoid any subjectivity in the assessment and detailed standard operation procedures were developed and followed at all times [21]. The facility assessments involved direct observations and interviews with health care providers and women. The following scoring methods were used [21]: If verification is possible by means of observation, no further triangulation is required. The relevant verification criterion is considered as “yes” if the practice/skill observed is being correctly performed on the women. If observation is not possible, then go for further triangulation methods as below;

- For case records, 50% or more of checked case records should indicate that the practice is performed at the facility to be considered as “yes”
- For providers’ interviews, 100% provider(s) interviewed should correctly respond for
  the verification criteria to be considered as “yes”
- For mothers’ interviews, 50% or more of mothers should provide a response indicating that the practice is being performed by providers

**Table 3:** Content of facility assessment tool

a. The availability of drugs, supplies and functional equipment in the labour room was checked by reviewing the stock register and interviewing the labour room incharge b. The labour room environment was visually assessed for adequate space, organization to ensure movement, seamless and uninterrupted working of staff and client privacy using a structured tool which has 7 standards and 28 verification criteria c. Adherence to evidence-based practices during the intrapartum and immediate postpartum period was assessed against 19 standards for care and 131 verification criteria, based on: 1) direct observation of providers during skills demonstrations on mannequins or during provision of actual care; 2) hospital record reviews; 3) provider interviews to assess knowledge; 4) client interviews; and 5) physical verification of supplies	

For a practice to be considered as “yes” in the response column, all the verification criteria under that practice should have a score of “yes” in the score column.

A standard will be scored either 0 or 1 depending on the responses. If the responses of all practices performed under a standard are “yes”, then the standard will score 1 point. If one or more responses for practices under a standard is “no”, the standard scores 0 points.

### Secondary data

#### Service statistics

This comprised the aggregate data submitted by public health facilities on key service delivery indicators to the Pregnancy, Child Tracking & Health Services Management System (PCTS) of Government of Rajasthan.

#### Monthly progress report 

Maternal and Newborn Complications Identification and Referral Monitoring System. Since, there was no formal mechanism in the government systems to monitor childbirth and newborn related complications and management, a monthly complication reporting format was introduced in all intervention facilities. The complication reporting format captured data on key maternal and newborn health indicators from the labour room, postpartum ward, Sick Newborn Care Unit, admissions and discharge department of the facility. This format captured data on total deliveries, type of deliveries, fresh stillbirths, preterm births, maternal and neonatal death, maternal complications (pre-eclampsia, eclampsia, sepsis, postpartum hemorrhage), neonatal complications (neonatal asphyxia and sepsis), and refer-in and refer-out data for maternal and neonatal complications. One nodal person, preferably a labour room staff, was selected from each intervention facility to act as the key contact person for this initiative. Data was validated regularly by the program team.

In order to ensure high quality of data, all program officers involved in data collection were trained before initiating data collection. Additionally, the program team conducted routine data quality checks using a data quality assurance toolkit [22].

Data from periodic assessments were entered in CS Pro [23] and dashboards were generated for timely data-driven decision-making.

#### Data analysis

In this manuscript, we have used data from June 2015 till December 2019 for competency of providers to perform evidence-based practices, availability of essential resources, labour room standards, adherence to evidence-based clinical standards, as well as the identification and management of maternal and neonatal complications. All these data were collected by Jhpiego staff who have medical background with several years of experience in implementation of maternal and child health programs in the state of Rajasthan. For stillbirths, we used PCTS data from March 2015 till March 2020 as it was reported in the Government of Rajasthan’s health management information system (PCTS).

## Results

### Program reach

During the program implementation period there were 17,94,249 deliveries, this accounts for 70% institutional deliveries in intervention districts. The staff in our intervention facilities were able to identify 37,565 maternal and 18,588 neonatal complications and referred 15,359 maternal and 7,416 neonatal complications.

### Strengthening the skills and competency of providers to perform evidence-based practices

Overall, 3,757 providers were trained from 525 facilities across Rajasthan state; providers included 77% staff nurses (2,899), 21% doctors (780) and ~ 2% lady health visitors and other staff. As a result of these trainings, there was a considerable increase in competency of health care providers in active management of third stage of labour (5% vs 92%), newborn resuscitation (3% vs 92%), management of antenatal complications (9% vs 85%) and management of postnatal complications (19% vs 77%).

### Improving the availability of essential supplies and commodities in the labour room and the postpartum wards

The availability of all 26 essential supplies and commodities in the labour room and postpartum wards significantly improved from 73 to 96% during program implementation (Table S1). At the beginning of the program, facilities had on average 19 essential supplies and commodities, while in the most recent assessment it increased to 25.

### Improving labour room standards

The analysis of labour room standards revealed that 29% of the intervention facilities met all 7 standards at the beginning of the program, compared to 71% of facilities in the latest assessment. It was also observed that the average number of labour room standards met increased from 2 to 5 during the course of the program (Table S2).

### Improving adherence to evidence-based clinical standards

The analysis of adherence to evidence-based clinical standards showed considerable improvement in all 19 standards over the program implementation period. The average number of standards met increased from 2 to 12 during the implementation period (Table S3). The standards that demonstrated maximum improvement over the course of the program were: pre-term delivery assessment (4% vs. 80%), provision of immediate newborn care (9% vs. 76%), active management of the third stage of labour (19% vs. 89%) and identification and management of severe pre-eclampsia/eclampsia (5% vs. 69%).

### Improvement in maternal and neonatal outcomes

#### Improved management of complications

**Fig. 3:**
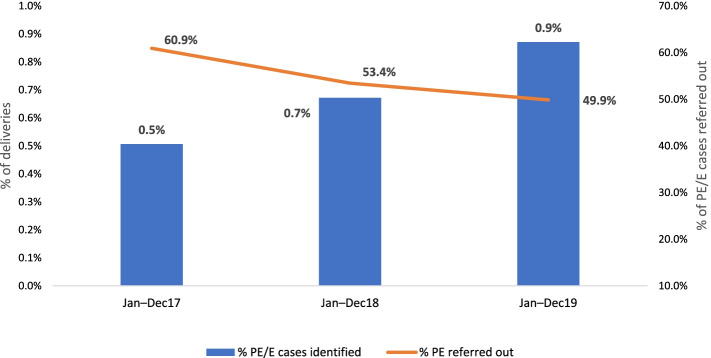
Trend of pre-eclampsia/eclampsia (PE/E) identification and referral out

Secondary analysis of data from complication reporting formats at Dakshata intervention facilities revealed 11 percentage points reduction in proportion of pre-eclampsia/eclampsia referred out during the time of childbirth (from 60.9% in January-December 2017 to 45.9% in January-December 2019) (Fig. 3).

**Fig. 4:**
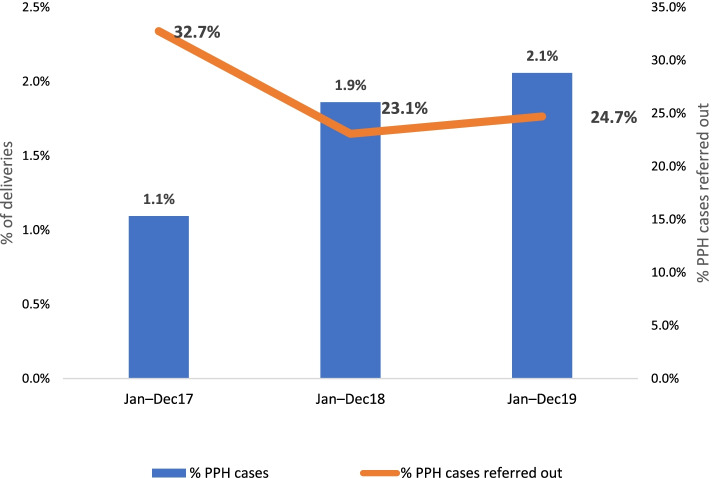
Trend of postpartum hemorrhage (PPH) identification and referral

**Fig. 5:**
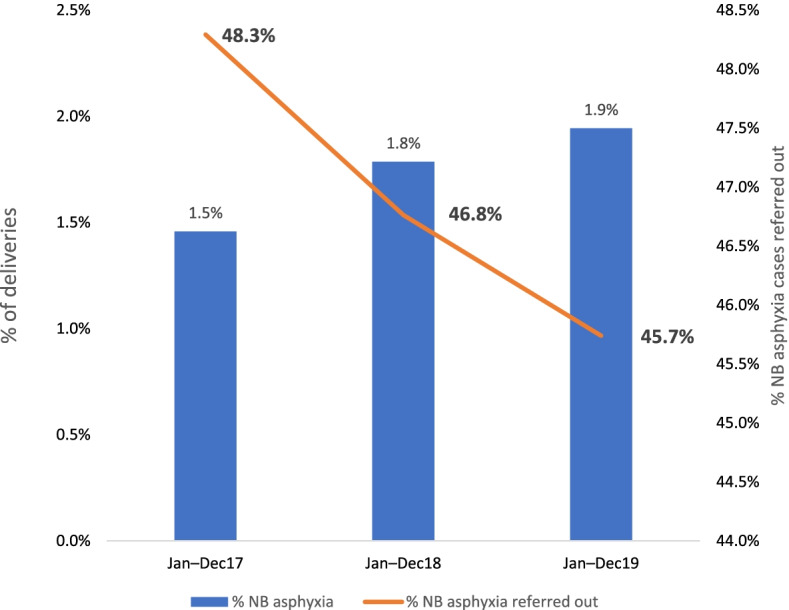
Trend of newborn asphyxia identification and referral out

The proportion of postpartum haemorrhage referrals decreased by 8 percentage points (from 32.7% in January-December 2017 to 24.7% in January-December 2019) (Fig. 4) and the proportion of newborn asphyxia referrals decreased by 3 percentage points (from 48.3% in January-December 2017 to 45.7% in January-December 2019) (Fig. 5).

### Reduction in stillbirth rate

**Fig. 6:**
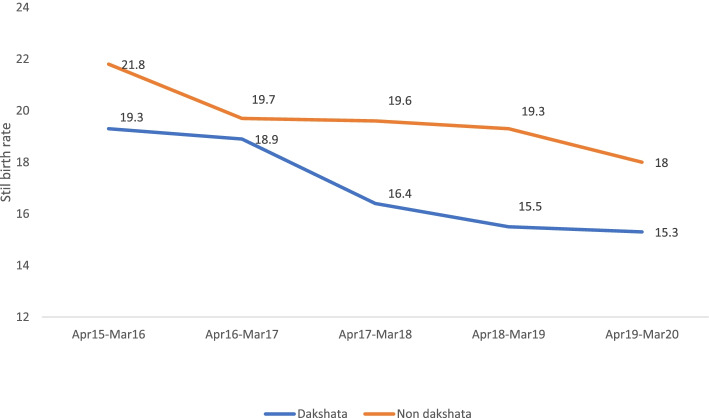
Trend of stillbirth rate (per 1000 live birth) in Dakshata facilities versus non Dakshata facilities

Similarly, PCTS data revealed a relatively greater reduction in stillbirth rates at Dakshata intervention facilities (19.3 vs 15.3) compared to non Dakshata facilities (21.8 vs 18) (Fig. 6).

## Discussion

Our findings are indicative that capacity building, mentorship support, focusing on high delivery load facilities, utilization of contextualized SCC, data-driven decision-making and government ownership resulted in improved service delivery.

Global evidence suggests that programs that only focus on capacity building of health care providers or targeting supply availability are insufficient to improve quality of care and outcomes [24]. It has been also proven that interventions that introduced checklists and in-person supervision are more effective in improving adherence to evidence-based standards and outcomes [25–27].

Capacity building of service providers to perform evidence-based practices was one of the main pillars of the Dakshata program. The training package included highly interactive “skills and drills” and “team training” approaches. The studies conducted across the globe indicates that this approach improves communication, coordination and cooperation among various health care cadre [28] and increases adherence to clinical standards [29]. However, the role of this approach in improving clinical outcomes is not clear [30].

The trainings were followed by mentorship activities for ensuring transfer of learnings into practice and availability of essential supplies and commodities. Previous studies conducted in Rajasthan [13], Uttar Pradesh [27] and Karnataka [31] states of India have highlighted the importance of mentorship and supportive supervision in improving adherence to evidence-based practices. In our study, obstetric simulation drills were conducted during structured MSVs in order to develop the facility readiness for the management of complications. Simulation is a powerful methodology of adult learning with the benefit of allowing for mistakes to occur without harm to a real patient [32, 33]. These simulation drills allowed providers to get acquainted with delivery room environment and equipment and also helped in improving communication among team members. Debriefing followed by simulation gave participants an opportunity to discuss the challenges faced and ways to address them.

Even structured MSVs package are not sufficient as providers have different learning needs and variable knowledge retention power. Besides, some of the practices are linked with providers’ perception and motivation to adhere to them. It is unrealistic to assume that providers’ behaviors, that developed during years of service tenure, will be changed in terms of adherence to practices with only capacity building and a few mentoring and support visits. Studies have demonstrated waning knowledge retention by 6 months following completion of the neonatal resuscitation program course [34, 35]. Continuous handholding seems to pave the way for positive outcomes during the course of Dakshata implementation. While the program team was able to capture practices with low adherence by using periodic assessments, it was difficult to track/identify low performers. Therefore, avoiding complacency and institutionalizing a mechanism for periodic assessment of competency and knowledge was of the utmost importance in this quality improvement initiative. This approach allowed the program team to further customize their focused mentoring approach to target poor performers and practices with low adherence.

As a part of improving evidence-based decision making, a standardized labour room case sheet was developed based on existing records in the facilities. However, as the program advanced, it became important to have data on process of care and data visualization (dashboards) for effective program implementation. Therefore, program team initiated the development of the Maternity Wing Management Information System (MWMIS) in which client case records were digitized, thus generating dashboards for district and state-level officials which could be utilized for effective program review. However, while MWMIS was useful for monitoring and tracking of labour room practices and outcomes, it was not serving the purpose of providing real-time data for action. Therefore, Jhpiego worked with donors to develop an Android-based clinical decision support system. MWMIS has been replaced by a real-time “Prasav Watch” (Labor Watch) application across the entire state of Rajasthan.

The Government of Rajasthan scaled up Jhpiego’s approach for sustaining quality improvement activities and hired dedicated Dakshata mentors. An induction package for Dakshata mentors were developed and a mechanism of 15 days mandatory internship for all newly joined mentors were ensured. However, it was challenging to monitor these mentors and track their progress. In order to sustain the quality improvement efforts in a comprehensive manner, an Android-based mobile application (the Dakshata Mentor App) was developed for mentors to enter the data of each facility visit, under domains such as training status, resource availability, labour room environment, adherence to practices and topics for which mentoring was provided. The data from this app are being used by the state for rapid and effective real-time monitoring of labour rooms.

Government ownership is not only critical for ensuring the sustainability of the program but also one of the key driving factors for effective continuity of the program. The program team engaged with government officials at all levels—facility, district and state level. After completion of any activity, whether it was an MSV or a periodic assessment, the team worked with facility in-charges to do a bottleneck analysis and developed an action plan. Some of the issues (shortage of staff, availability of essential commodities), which could not be addressed at the facility level, were discussed during district review meetings and addressed. Since the initiation of the program, the state has budgeted for all program activities in the annual project implementation plans under the National Health Mission, for training, strengthening of labour room infrastructure, procurement of essential resources, as well as recruiting dedicated Dakshata mentors to sustain the program in respective districts. The state conducted regular review meetings with facility in-charges and district-level officials in order to ensure that identified gaps are being addressed in a timely manner and to hold poor-performing facilities accountable. Thus, the steadfast commitment of the state has been instrumental in the smooth implementation of the program.

Additionally, based on independent midline evaluation results and recommendations, key corrective actions were taken that resulted in considerable improvement (Table 4).

**Table 4:** Role of Independent evaluation

In Rajasthan, the evaluation partner collected data for 3 time points, i.e., Baseline (Oct’17-Feb’18), Midline (May’18-Sep’18) and Endline (May’19-Sep’19). Based on Midline evaluation assessment, the following corrective actions were undertaken:	
• Engaged state for addressing inadequate number of nursing staff in labour rooms by sufficient deployment of staff and initiation of recruitment process of 6,500 new nursing staff • Fixed accountability of providers and labour room management information system data used for decision-making • Initiated special pilots to address “sticky practices” like hand-hygiene and post-delivery practices	

It is important to mention that Dakshata program targeted 202 (19%) high delivery load public facilities. This approach supported improving care provided to 70% of deliveries taking place in public facilities of selected districts in a short time period, thereby contributing to better maternal and neonatal outcomes.

## Challenges

The multidisciplinary nature of the labour rooms, the complexity of task involved and lack of tested mechanisms to track measurable processes and outcomes possess challenges [36]. Despite the considerable changes, the following challenges are important to mention:

### Staff shortages and turnover

Frequent rotation of trained human resources from labour rooms to other wards of the facilities led to another challenge of ensuring trained and adequately skilled staff at labour room. This required extra efforts by the mentor to train newly transferred staff on the clinical guidelines and processes in the labour room. In order to minimize this hindrance, the state has issued directives on the non-rotation of trained competent staff in the intervention facilities. Program team also developed a nurse duty roster in MWMIS to allow state officials to track the status of trained human resources in labour rooms. Additionally, the team worked with the state government to identify the deficiency in availability of staff, as per norms, and address them. Based on the requirement, state initiated the process of recruiting additional nursing staff.

### Improper data recording and reporting

The Government of Rajasthan worked to streamline the data recording process by standardizing the labour room case sheet. However, lack of awareness among nursing staff regarding importance of proper data recording and shortage of nursing staff at labour room were major hindrance in getting quality data to guide decision-making processes. To tackle this issue, the MWMIS tool was developed. Additionally, suboptimal entry of case sheets data into MWMIS by the data entry operators was another bottleneck. As these issues can affect the utilization of this data for decision-making, the state has been focused on orienting the service providers on ensuring the completeness of case sheets and it has been focused on tracking the performance of the data entry operators.

### Addressing behavioral factors affecting uptake of practices

Some of the essential practices showed poor adherence, which we termed them as sticky practices, despite repeated mentorship visits, including hand hygiene, plotting of partograph, newborn resuscitation, post neonatal monitoring and counselling. These sticky practices varied across the facilities. Facilities with higher delivery loads and acute shortage of staff are more likely to omit most of these practices. Practices which showed immediate gratification, in terms of life saved or complication averted, were quickly adopt by providers. On the other hand, practice which do not show any immediate harm or benefits, like vital measurements, are difficult to achieve. There are certain practices for which there are not ample opportunities to practice (e.g. newborn resuscitation) that are again difficult to demonstrate. Most importantly, practices which providers have been observing since their initial days are difficult to stop, such as augmentation of labour, and will require focused efforts and close supervision from the administrative authorities. There are many determinants of service providers’ adherence to evidence-based practices, such as knowledge about the importance of particular practices, providers’ attitudes and personal motivations and immediate results observed after performing the practices.

### Wide variation in ownership at the facility and by district-level managers

Being a government-led initiative, the implementation of Dakshata program requires ownership and accountability at all levels of the health system. Starting from district-level sensitization meeting to organizing district-/state-level review meeting, program team worked to bring ownership at all levels. Despite best efforts, some of the facilities did not show expected improvement. At the end of each periodic assessment, the team did bottleneck analysis and developed an action plan which pointed towards lack of ownership by facility in-charges as an important factor for poor performance of some facilities. To address this issue, the program team identified a champion from the existing staff working at the facility level based on their ability and motivation to bring changes and communication skills. These champions were later designated as the nodal person for the Dakshata program at their facility and have been a key step forward in this regard. Nodal person helped in ensuring supplies at point of use and bring infrastructural changes to labour rooms.

## Conclusion

The Dakshata program succeeded in improving the quality of care provided during labour and immediately after delivery in the state of Rajasthan. Despite this intervention being scaled up across the country, there is no systematic analysis of weakness, opportunities and the learnings from different states across India. It is, therefore, imperative to conduct assessments and data reviews to guide future programming for improving maternal and neonatal outcomes.

## Supplementary Information

**Additional file1: Table S1.** The availability of resources in labour room and postpartum wards (*n*=202). **Table S2.** Labour room standards (*n*=202). **Table S3.** Providers’ adherence to key clinical standards.

## Data Availability

Qualified researchers may request data access by emailing the corresponding author. In such an event, the researchers will consult with the Government of Rajasthan's Department of Medical, Health and Family Welfare before providing data access to the concerned parties.

## Abbreviations

CIFF: Children’s Investment Fund Foundation
MoHFW: Ministry of Health and Family Welfare
MSV: Mentorship and support visits
MWMIS: Maternity Wing Management Information System (MWMIS)
PA: Periodic assessments
PCTS: Pregnancy, Child Tracking & Health Services Management System
RA: Rapid assessment
SDG: Sustainable Development Goals
SCC: Safe Childbirth Checklist
