# Supplementary material for: Improving quality of intrapartum and immediate postpartum care in public facilities: experiences and lessons learned from Rajasthan state, India
Source: BMC Pregnancy Childbirth. 2022 Jul 23;22:586. doi: 10.1186/s12884-022-04888-5 (PMC9308226; doi:10.1186/s12884-022-04888-5)
Supplement: Supplementary file 1 — Additional file1: Table S1. The availability of resources in labour room and postpartum wards (n=202). Table S2. Labour room standards (n=202). Table S3. Providers’ adherence to key clinical standards. [file 12884_2022_4888_MOESM1_ESM.docx]

Supplementary table 1: The availability of resources in labour room and postpartum wards (n=202)

| **S.No.** | **Resources** | **RA Score (June–August 2015)** | **Latest PA Score (May-– 2019)** | **% RA** | **% Latest PA** |
| --- | --- | --- | --- | --- | --- |
| 1 | Magnesium sulphate (at least 20 vials) | 125 | 198 | 62% | 98% |
| 2 | Antibiotics for mother | 150 | 200 | 74% | 99% |
| 3 | Antibiotics for baby | 142 | 197 | 70% | 98% |
| 4 | Oxytocin (5/10 IU per ml) | 187 | 197 | 93% | 98% |
| 5 | Vitamin K (1mg/ml or 1 mg/0.5 ml) | 134 | 196 | 66% | 97% |
| 6 | IV fluids | 191 | 199 | 95% | 99% |
| 7 | Antiretrovirals | 26 | 151 | 13% | 75% |
| 8 | Soap & running water | 163 | 197 | 81% | 98% |
| 9 | Gloves | 191 | 201 | 95% | 100% |
| 10 | Uristick (for proteinuria and glucose) | 51 | 193 | 25% | 96% |
| 11 | Partograph | 167 | 200 | 83% | 99% |
| 12 | Cord clamps | 184 | 198 | 91% | 98% |
| 13 | Sterile scissors | 122 | 164 | 60% | 81% |
| 14 | Pads | 167 | 136 | 83% | 67% |
| 15 | Towels for receiving newborns | 81 | 172 | 40% | 85% |
| 16 | Disposable syringes and disposable needles | 196 | 202 | 97% | 100% |
| 17 | IV Sets | 196 | 202 | 97% | 100% |
| 18 | Corticosteroids (Inj. Dexamethason) | 194 | 202 | 96% | 100% |
| 19 | Ambu bag for babies | 112 | 199 | 55% | 99% |
| 20 | BP apparatus | 141 | 198 | 70% | 98% |
| 21 | Stethoscope | 134 | 196 | 66% | 97% |
| 22 | Thermometer | 134 | 191 | 66% | 95% |
| 23 | Mucus extractor | 152 | 198 | 75% | 98% |
| 24 | Suction device | 188 | 200 | 93% | 99% |
| 25 | Functional radiant warmer | 175 | 201 | 87% | 100% |
| 26 | Protocol posters displayed | 132 | 182 | 65% | 90% |
| Overall out of 26 resources | | 19 | 25 | 73% | 96% |
| RA, rapid assessment; PA, periodic assessment | | | | | |

Supplementary table 2: Labour room standards (n=202)

| **Labour Room Standards** | | **RA Score (June–August 2015)** | **Latest PA Score (May–August 2019)** | **% RA** | **% Latest PA** |
| --- | --- | --- | --- | --- | --- |
| 1 | Adequate provision of privacy | **39** | **151** | **19%** | **75%** |
| 2 | Good ambience | **138** | **181** | **68%** | **90%** |
| 3 | Adequate ventilation and lighting | **37** | **153** | **18%** | **76%** |
| 4 | Adequacy of labour and delivery facilities | **2** | **113** | **1%** | **56%** |
| 5 | Appropriate space management | **10** | **117** | **5%** | **58%** |
| 6 | Accessibility of functional washroom | **46** | **105** | **23%** | **52%** |
| 7 | Availability of functional hand washing station in labour room | **101** | **181** | **50%** | **90%** |
| **Overall out of 7 standards** | | **2** | **5** | **29%** | **71%** |
| RA, rapid assessment; PA, periodic assessment | | | | | |

Supplementary table 3: Providers’ adherence to key clinical standards (n=202)

| **Standard No.** | **Practice Assessment** | **RA Score (June -August 2015)** | **Latest PA Score (May-August 2019)** | **% RA** | **% Latest PA** |
| --- | --- | --- | --- | --- | --- |
| 1 | Appropriate and adequate assessment of clinical condition of pregnant woman and fetus at the time of admission | 5 | 80 | **2%** | **40%** |
| 2 | Appropriate internal examination | 8 | 89 | **4%** | **44%** |
| 3 | Identification and management of HIV in pregnant woman and newborn | 11 | 90 | **5%** | **45%** |
| 4 | Identification and management of infection in pregnant women | 0 | 52 | **0%** | **26%** |
| 5 | Identification of conditions leading to preterm delivery and facilitation of preventive care | 9 | 161 | **4%** | **80%** |
| 6 | Identification and management of severe pre-eclampsia/eclampsia | 10 | 140 | **5%** | **69%** |
| 7 | Monitoring the progress of labour and adjustment of care accordingly (partograph) | 15 | 83 | **7%** | **41%** |
| 8 | Ensuring respectful and supportive care | 55 | 142 | **27%** | **70%** |
| 9 | Preparation for safe care during delivery | 9 | 135 | **4%** | **67%** |
| 10 | Assistance in safe and clean birth | 8 | 127 | **4%** | **63%** |
| 11 | Performance of immediate newborn care | 18 | 153 | **9%** | **76%** |
| 12 | Performance of newborn resuscitation (if baby does not cry immediately after birth) | 9 | 112 | **4%** | **55%** |
| 13 | Performance of AMTSL | 39 | 179 | **19%** | **89%** |
| 14 | Identification and management of postpartum haemorrhage | 61 | 168 | **30%** | **83%** |
| 15 | Assessment of condition of newborn and mother before shifting them from labour room | 5 | 41 | **2%** | **20%** |
| 16 | Ensuring exclusive and on-demand breastfeeding | 106 | 190 | **52%** | **94%** |
| 17 | Ensuring care of newborn with small size at birth | 39 | 152 | **19%** | **75%** |
| 18 | Counselling at discharge | 81 | 156 | **40%** | **77%** |
| 19 | Adherence to universal infection prevention protocols | 9 | 87 | **4%** | **43%** |
| **Overall out of 19 standards** | | **2** | **12** | **11%** | **63%** |
| RA, rapid assessment; PA, periodic assessment; AMTSL, active management of third state of labour | | | | | |
